# Interdisciplinary Orthodontic–Endodontic Management of Complex Dental Trauma Involving Three Permanent Anterior Teeth: A Clinical Case Report

**DOI:** 10.3390/dj14050288

**Published:** 2026-05-11

**Authors:** Ioannis P. Zogakis, Chrysanthi Anagnostou, Panagiotis Zogakis

**Affiliations:** 1 Department of Orthodontics, Faculty of Health Sciences, School of Dentistry, Aristotle University of Thessaloniki, 54124 Thessaloniki, Greece; 2 Department of Hygiene, Social-Preventive Medicine and Medical Statistics, Faculty of Health Sciences, School of Medicine, Aristotle University of Thessaloniki, 54124 Thessaloniki, Greece; 3 Department of Endodontology, Faculty of Health Sciences, School of Dentistry, Aristotle University of Thessaloniki, 54124 Thessaloniki, Greece

**Keywords:** dental trauma, traumatic luxation, intrusion, extrusion

## Abstract

**Background and Clinical Significance**: Dental trauma presents a considerable challenge for clinicians due to the diverse and complex effects on teeth. Effective management often requires the collaboration of multiple specialists, including endodontists and orthodontists. **Case Presentation**: This case report presents the interdisciplinary management of a complex dental trauma case involving three permanent maxillary anterior teeth. Unlike most reports of dental trauma, this case includes complete pre-trauma diagnostic records, providing valuable baseline information for treatment planning and outcome assessment. A 15-year-old female patient was examined in the orthodontic clinic, with comprehensive diagnostic records being obtained at the initial visit. Before the commencement of active orthodontic therapy, the patient experienced an extraoral traumatic incident. Clinical and radiographic assessment revealed concussion and traumatic mobility of the upper right canine, intrusive luxation of the upper right lateral incisor and extrusive luxation with increased mobility of the upper right central incisor. Taking into consideration treatment alternatives, an orthodontic–endodontic approach was preferred. **Conclusions**: The successful management of complex dental trauma affecting multiple permanent teeth requires interdisciplinary collaboration. The clinical significance lies in the potential long-term consequences on both tooth function and aesthetics, which can impact patient well-being.

## 1. Introduction and Clinical Significance

Dental trauma is a prevalent health issue, affecting both permanent and deciduous teeth. Research shows that 10.5% to 17.3% of the general population is affected by dental trauma [1,2], with a prevalence of 13% to 25% in the 8–15 age group and no significant gender differences [1,3]. Central incisors are the most frequently injured teeth (60%), followed by lateral incisors, while from a malocclusion perspective, Angle class II malocclusion is a key risk factor for dental trauma [1,4,5,6].

Tooth luxation, accounting for 18–33% of dental trauma in permanent teeth, often results in significant damage to the pulp and surrounding tissues. It involves the displacement of a tooth from its socket without complete avulsion, caused by acute trauma [1]. Intrusive luxation, the most severe form, involves apical displacement of the tooth, leading to a visually shorter crown compared to the contralateral tooth. This type of injury is associated with a poor prognosis [7]. Extrusive luxation, or partial avulsion, causes the tooth to shift, often palatally, damaging the vascular-nervous bundle and stretching the periodontal fibers [8]. This condition is characterized by elongation of the tooth, increased mobility and occlusal interference [9]. Lateral luxation occurs due to lateral forces, frequently resulting in damage to the vascular-nerve bundle and may be accompanied by fractures of the buccal bone cortex or compression of the periodontal tissues [10].

Dental trauma presents a significant challenge for clinicians due to the complex and varied effects that such injuries may exert on teeth. Effective management often requires the combined expertise of multiple specialists, including endodontists and orthodontists. One of the most severe forms of dental trauma is intrusive luxation, characterized by the axial displacement of the tooth into the alveolar bone. This injury often results in extensive damage to the periodontal ligament, cementum and pulp tissue, making its management particularly complex and prognostically challenging. If a tooth sustaining intrusive luxation is not treated in a timely and appropriate manner, it may compromise the integrity of the dentition and prove unsuitable as a stable abutment for future prosthetic rehabilitation. In growing individuals, the intruded tooth is likely to become progressively infraoccluded in comparison with adjacent teeth, while the associated alveolar bone may exhibit deficient growth and reduced volume relative to the bone surrounding unaffected teeth [11].

The clinical significance of dental trauma extends beyond the immediate injury, as it can lead to long-term consequences that affect both tooth function and aesthetics. Functional complications may include issues such as compromised occlusion, impaired masticatory ability, or even tooth loss, all of which can affect a patient’s daily life and overall oral health. Aesthetic concerns, such as tooth discoloration and malalignment can significantly impact a patient’s self-esteem and social interactions [12]. Additionally, untreated or poorly managed trauma may lead to complications like pulp necrosis, root resorption, or chronic pain, requiring more extensive and costly treatments in the future. As such, timely and comprehensive management is essential to minimize these risks and preserve both the function and appearance of the teeth, thereby ensuring optimal patient well-being.

The present report describes the interdisciplinary orthodontic–endodontic management of a complex case of dental trauma involving three permanent anterior teeth. The primary objectives of the orthodontic intervention were the repositioning of the affected teeth into their normal positions and the facilitation of the required root canal treatments, with the ultimate goal of ensuring functional stability and long-term tooth longevity. A distinguishing feature of this case is the availability of initial records obtained before the injury, offering a rare perspective on the impact of trauma and subsequent management.

## 2. Case Presentation

### 2.1. Case History

**Figure 1:**
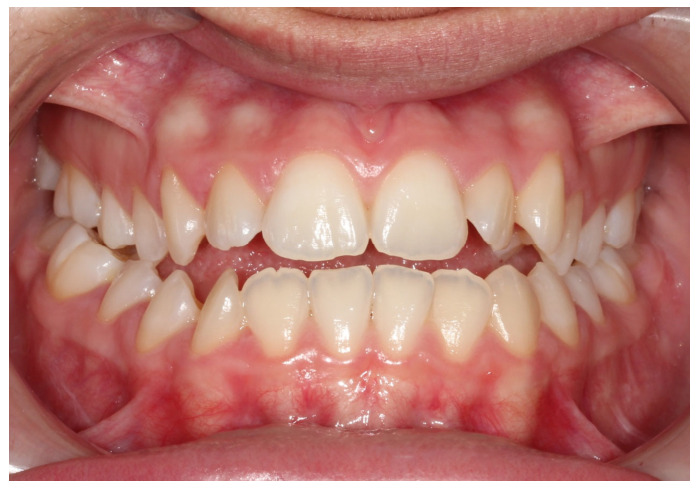
Frontal intraoral view in maximum intercuspation prior to traumatic injury.

**Figure 2:**
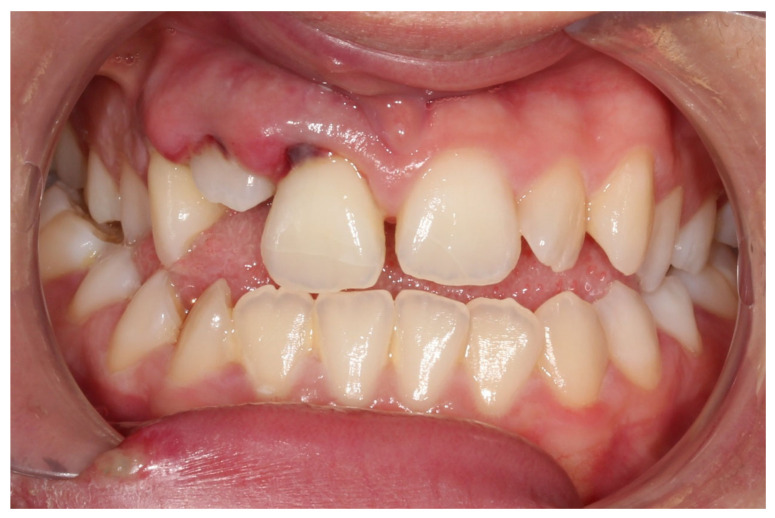
Frontal intraoral view in maximum intercuspation after traumatic injury.

**Figure 3:**
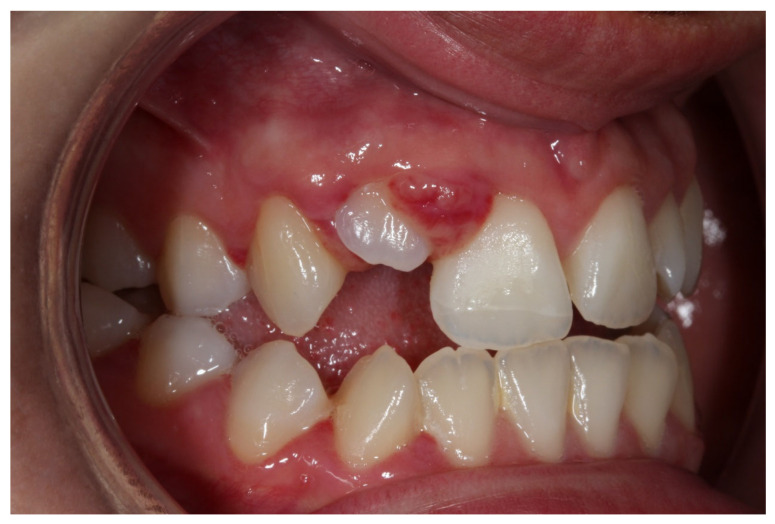
Close-up intraoral view of the involved teeth after traumatic injury.

**Figure 4:**
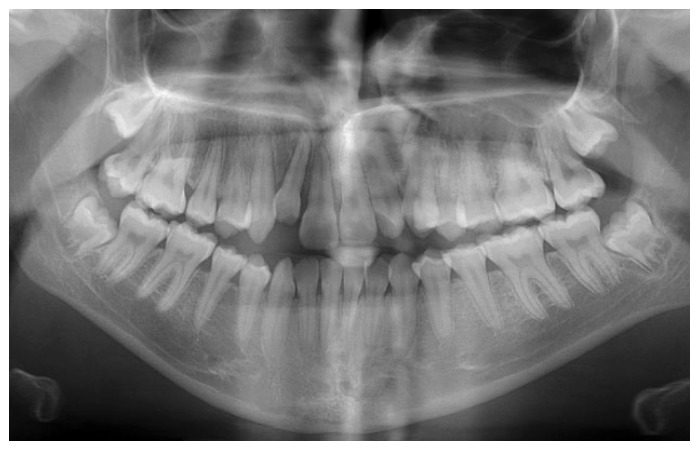
Panoramic radiograph after traumatic injury.

**Figure 5:**
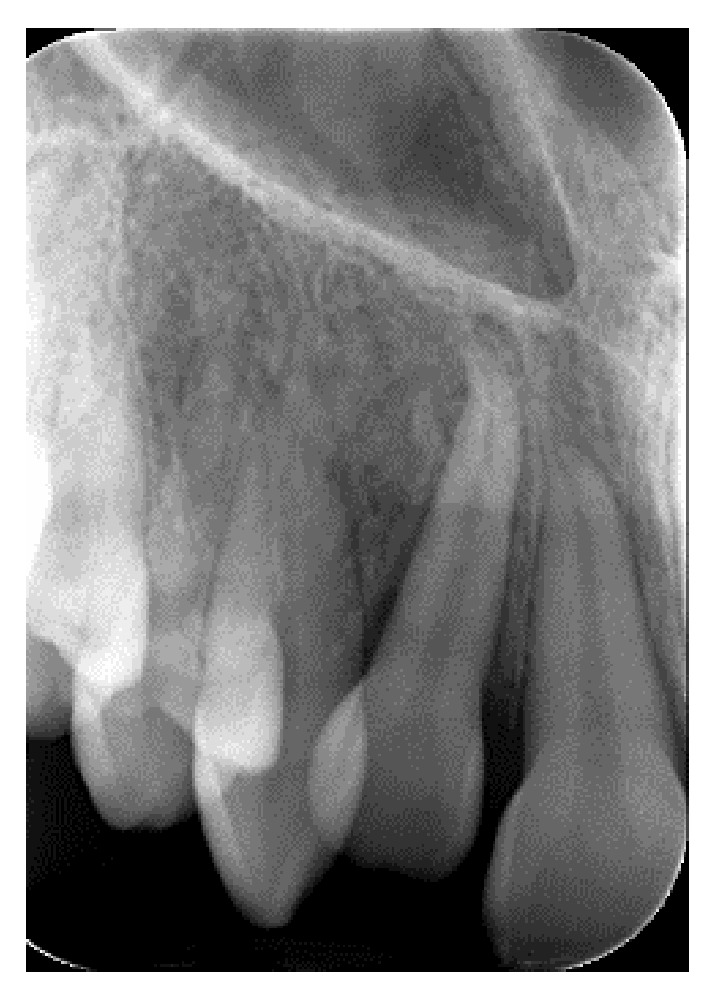
Periapical radiograph after traumatic injury.

A systemically healthy (ASA I) 15-year-old female patient, with no history of dental trauma, visited the orthodontic clinic seeking orthodontic treatment and initial diagnostic records were obtained (Figure 1). The orthodontic assessment identified a Class III malocclusion according to Angle’s classification, mild crowding in both the maxillary and mandibular arches and an anterior open bite. Prior to the initiation of active orthodontic therapy, the patient sustained an extraoral traumatic injury at a playground. Clinical and radiographic examination revealed the following findings: (a) concussion of the upper right canine, (b) acute intrusive luxation of upper right lateral incisor, and (c) extrusive luxation accompanied by increased mobility of the upper right central incisor (Figure 2, Figure 3 and Figure 4). The radiographic examination demonstrated complete root formation. The incisal edge of the intruded tooth was positioned at the level of the cemento-enamel junction of the adjacent teeth (Figure 5). 

### 2.2. Treatment Considerations—Alternatives

Key treatment considerations in this case included determining the optimal timing for initiating orthodontic intervention and selecting an appropriate anchorage strategy. In terms of optimal timing, one should consider that a prolonged delay, as a treatment approach, may result in ankylosis of the intruded tooth, rendering subsequent extrusion forces ineffective. Conversely, as an alternative treatment plan, initiating traction immediately, before the periodontal fibers have healed and adequate periodontal support has been established, may compromise the tooth. The anchorage decision was particularly critical given that the teeth adjacent to the intruded tooth were also traumatized, with noticeable mobility, necessitating a cautious and biologically sensitive approach to effectively employ them as anchor units. Although various treatment modalities have been mentioned, ranging from removable appliances, direct bonding of rigid wire to anchored teeth with elastomeric force application, to self-supported labial arch on fixed molar bands [11], a simple, “straight wire” approach, was preferred in this case to achieve light continuous force application.

### 2.3. Management of the Case

**Figure 6:**
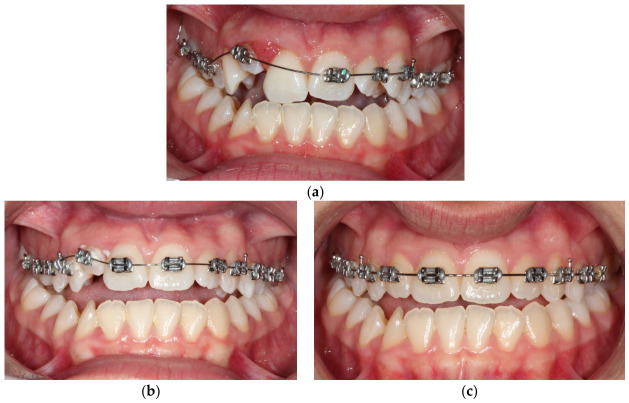
Treatment progress photographs: (**a**) Frontal intraoral view in maximum intercuspation after appliance bonding. The upper right central incisor was bypassed to reduce the force magnitude on adjacent teeth by increasing the inter-bracket distance. (**b**) Frontal intraoral view demonstrating treatment progress after two months with full engagement of a continuous Ni-Ti archwire. (**c**) Frontal intraoral view in maximum intercuspation before appliance debonding (pre-final stage), with alignment and leveling of the upper arch complete.

Orthodontic treatment of the maxillary dental arch commenced 3.5 weeks after the traumatic incident, with the bonding of pre-adjusted edgewise 22-slot brackets to the upper arch, and the archwire was partially engaged. Initially, a 0.012-inch nickel–titanium archwire was inserted to facilitate initial leveling and alignment. Two months later, once the mobility of the upper right central incisor has significantly decreased, a bracket was placed on its labial surface and the tooth was included in the appliance (Figure 6). Over the course of the treatment, the archwires were gradually replaced with progressively larger sizes. This sequence of wire changes was essential in achieving optimal alignment and leveling of the dentition. The patient attended regular follow-up appointments on a monthly basis, during which adjustments and activations of the appliance were performed to ensure continued progress in the treatment. For the correction of the intruded lateral incisor, forces were effectively applied through the main archwire, which were sufficient to facilitate both the extrusion and alignment of the tooth. The gradual changes in archwire size, in conjunction with the strategic force application, allowed for controlled movement of the lateral incisor, ensuring its optimal repositioning and integration within the occlusion. This approach contributed significantly to the overall treatment goals of achieving proper alignment and esthetic harmony in the upper arch. Regarding the upper right central and lateral incisor, pulp vitality testing with a cold spray at −20 °C elicited no response and an electric pulp test was also negative, as expected. Six months post-trauma, once the crown of the traumatized lateral incisor was sufficiently exposed to permit endodontic intervention, the patient was referred for endodontic treatment of the traumatized lateral and central incisor.

Following coronal access and measurements, root canal preparation was carried out using the step-back technique to shape the root canal system. The canals were then irrigated with 5.25% sodium hypochlorite to remove debris and disinfect the canal space. After cleaning, a calcium hydroxide-based intracanal dressing was applied to promote further disinfection and ensure complete healing of the periapical tissues. Following the dressing phase, the root canal was obturated using gutta-percha points in conjunction with an epoxy resin-based sealer to provide an impermeable seal and optimal root canal filling, employing the laterally condensed gutta-percha technique. Subsequently, the palatal surface was restored with a light-cured composite resin, chosen for its excellent bonding properties and aesthetic results. Final radiographs were taken to confirm complete obturation and proper sealing of the root canal system. The access cavity was sealed with composite resin, ensuring a durable, functional, and esthetic restoration.

**Figure 7:**
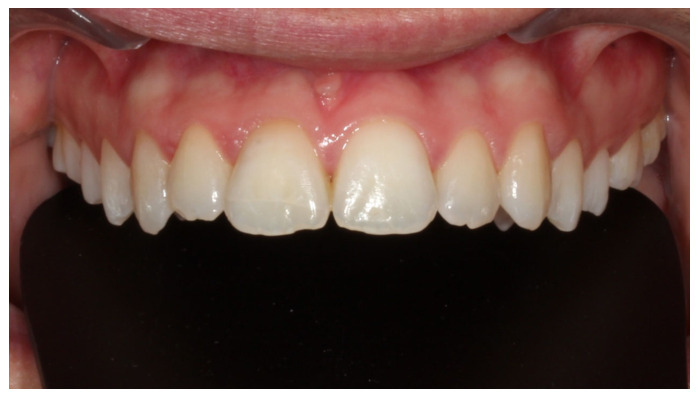
Frontal intraoral view of the upper teeth after appliances’ debonding.

**Figure 8:**
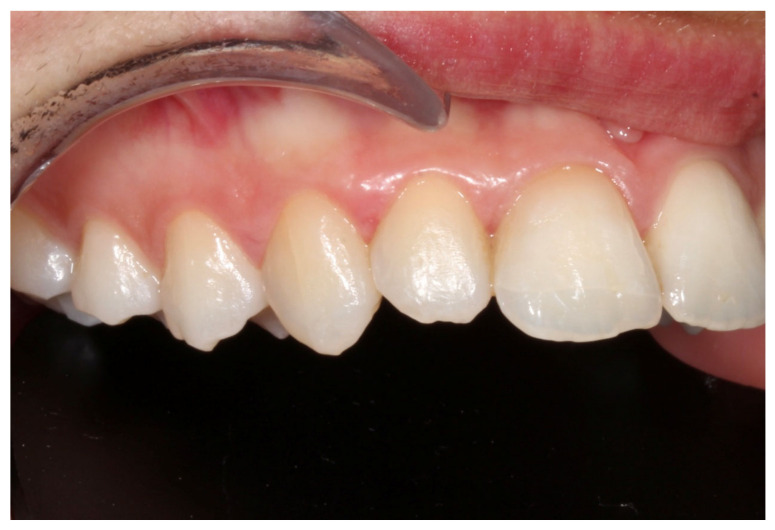
Close-up intraoral view of the involved teeth after appliances’ debonding.

Fixed orthodontic appliances were not placed in the lower dental arch, as planned initially and prior to trauma, due to a modification of treatment objectives following the traumatic injury. The total duration of the orthodontic treatment was 16 months. Fixed retainer was bonded to the palatal surfaces of the maxillary left central and lateral incisor and a thermoplastic clear retainer was also provided to preserve the achieved orthodontic alignment, with the patient be advised to use 12 h/day. Final treatment records are illustrated in Figure 7 and Figure 8.

**Figure 9:**
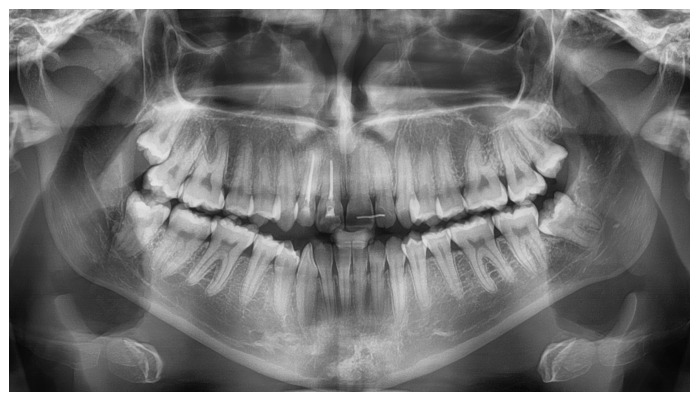
Panoramic radiograph 4 years after treatment completion.

**Figure 10:**
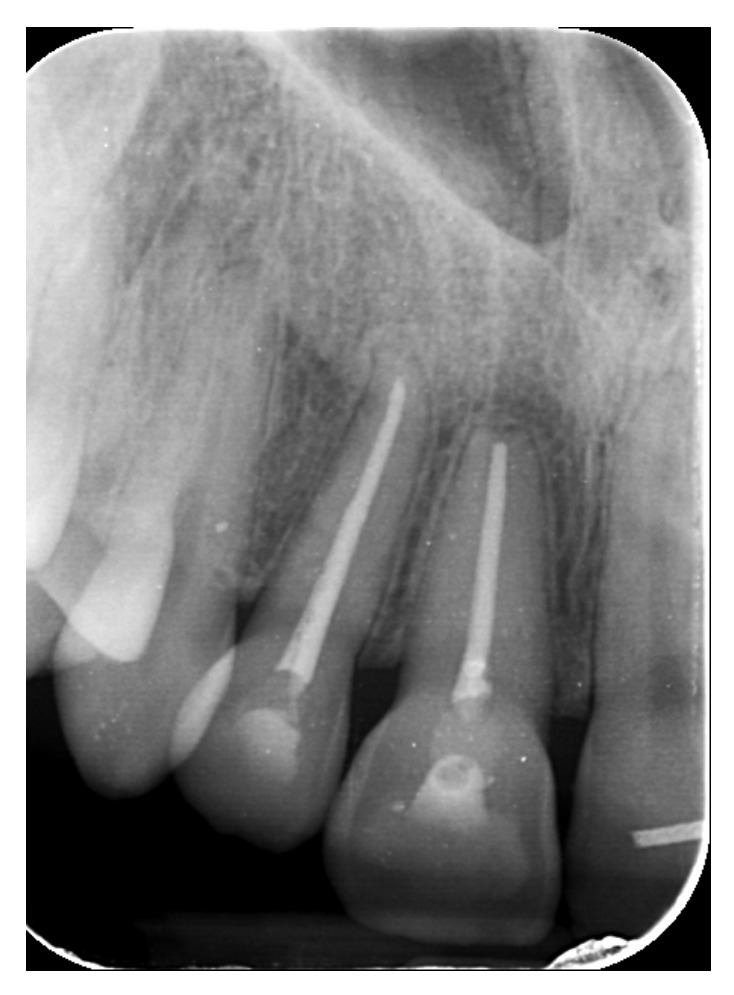
Periapical radiograph 4 years after treatment completion.

Semi-annual follow-up appointments were scheduled to ensure on-going monitoring and maintenance. In Figure 9 and Figure 10 radiographical follow-up is presented, conducted at 4 years after treatment completion. Radiographic examination revealed integrity of the lamina dura and absence of active inflammatory external or replacement resorption. Mild orthodontically induced root resorption can be noticed in the apical area of the upper right central incisor. The patient remained asymptomatic and expressed a high level of satisfaction with the outcomes of her treatment, noting that no visible signs of trauma remain. The successful restoration of both function and aesthetics has effectively eliminated any evidence of the injury, allowing the patient to regain confidence in her appearance. This positive result underscores the importance of timely, coordinated care and the expertise of the multidisciplinary team in achieving a favorable, long-term resolution of complex dental trauma. The absence of visible trauma not only reflects the clinical success of the interventions but also significantly contributes to the patient’s overall well-being and quality of life.

## 3. Discussion

The management of luxation injuries is dictated by the nature of the displacement: intrusive luxation can be managed via spontaneous re-eruption through manipulative or surgical repositioning, followed by splint stabilization or orthodontic intervention [13], whereas extrusive luxation may be addressed through manipulative or surgical repositioning with subsequent splinting or orthodontic therapy. The treatment of choice in this case was orthodontic reduction, as treatment alternatives are associated with certain limitations. Vertically displaced teeth may spontaneously re-erupt, regaining their original position, particularly if the root apex is open [14,15]. However, persistent intrusion after several weeks necessitates active repositioning [16,17]. On the other hand, successful immediate repositioning requires careful realignment and splint stabilization to allow healing of the periodontal and gingival fibers, with tooth prognosis depending on the extent of root resorption, the healing of pulp and periapical tissues after endodontic treatment and the re-establishment of periodontal attachment [11]. Orthodontic management was preferred in the presented case, taking into consideration the patient’s adolescent age, the closed root apices and the absence of prompt post-trauma care.

When employing fixed appliances to orthodontically manage intruded teeth, particular caution is essential [16]. Any extrusive tooth movement requires a stable resistance framework bonded to adjacent teeth to serve as a multi-unit anchorage system from which force can be applied to the intruded tooth, typically achieved through a combination of brackets and an archwire. However, because the neighboring teeth are often traumatized during the initial injury, using them as anchorage may risk further damage, even under minimal force [11].

If orthodontic forces are applied to an intruded tooth immediately after trauma, when few periodontal fibers remain intact, exfoliation is probable. Therefore, traction should be delayed allowing healing and partial reattachment of periodontal fibers, with the timing of orthodontic intervention being the focus of previous studies [18]. Successful reintegration of the tooth with bone depends on fiber healing, either alone or with surface resorption. Surface resorption allows reattachment through a normal periodontal ligament and new cementum, enabling orthodontic movement. In contrast, replacement resorption creates a direct bond between root and bone, preventing tooth movement. This ankylosis leaves the tooth permanently intruded [19], leading over time to infraocclusion and associated functional and esthetic problems. Furthermore, the periodontal implications of ankylosis are significant, as the absence of a functional periodontal ligament compromises the tooth’s ability to withstand occlusal forces, potentially leading to further bone loss and the deterioration of the surrounding soft tissue support. The lack of normal periodontal function also limits the potential for future orthodontic intervention or restoration of normal occlusion. In such cases, active extrusive forces are ineffective, as they are transferred to the anchoring teeth, potentially causing their unintended intrusion [11]. Literature suggests that a period of 2 to 4 weeks post-trauma is typically necessary to allow for sufficient fiber reattachment, minimizing the risk of complications and improving the prognosis for orthodontic correction [11]. Therefore, the timing of orthodontic intervention must be tailored to the individual case, taking into account the degree of trauma, the status of the periodontal ligament and the healing response.

In terms of the need for endodontic treatment, intrusion represents one of the most severe forms of luxation injury, resulting in pulp necrosis in approximately 96% of teeth with fully developed roots [20]. According to Chaushu et al., all intruded teeth with closed apices lost vitality irrespective of the extent of intrusion, and late complications, such as inflammatory root resorption, occurred in teeth with closed apices that did not receive immediate endodontic treatment [21]. Moreover, based on previous findings, maxillary incisors with a history of severe periodontal trauma demonstrate an increased risk of pulp necrosis during both orthodontic extrusion and intrusion compared with non-traumatized teeth [22,23]. Accordingly, timely endodontic treatment is essential to minimize the risk of inflammatory external root resorption. Generally, pulpal complications following luxation injuries in permanent teeth are largely determined by the type and direction of tooth displacement, the diameter of the apical foramen, and the presence of concomitant crown fractures. Pulp necrosis and pulp canal obliteration occur frequently, particularly in intruded, lateral, or extrusively luxated teeth and in teeth with closed apices, whereas pulp vitality is more likely preserved in teeth with open apices and without associated crown fractures [24].

Regarding periodontal implications, according to a recent study, in permanent teeth undergoing orthodontic extrusion after traumatic intrusion, periodontal healing was generally favorable, with most teeth showing functional recovery. Replacement root resorption or ankylosis was rare in teeth with immature roots, and inflammatory root resorption occurred in a minority of cases. In teeth with mature roots, adverse periodontal outcomes were slightly more common but still limited, and no significant differences were observed between treated and control teeth. Overall, orthodontic extrusion appears to support periodontal health, indicating that careful force management and timely intervention can help minimize complications [25].

The strengths of this case include the involvement of multiple traumatized permanent teeth that were successfully managed through a comprehensive and interdisciplinary treatment approach. Moreover, the availability of comprehensive pre-trauma records allowed for accurate diagnosis, comparison, and evaluation of treatment outcomes. The main limitation of this case report is the lack of statistical analysis, as it relies solely on descriptive data. To draw more reliable and generalizable conclusions, additional cases of a similar nature should be collected and analyzed systematically.

When treating dental trauma involving multiple teeth, a patient-centered approach is crucial for ensuring both effective care and optimal outcomes [26]. This approach considers not only the immediate clinical needs but also the emotional and psychological impact of the injury on the patient. Multiple tooth trauma often requires a multi-disciplinary treatment plan [27,28], involving specialists such as endodontists, orthodontists, and oral surgeons, to address the varying degrees of damage. From a patient perspective, the treatment process must be managed in a way that minimizes pain, anxiety and inconvenience while maximizing the long-term functionality and aesthetics of the teeth. In addition to restoring tooth function and appearance, it is important to provide clear communication, set realistic expectations, and offer support throughout the recovery process. Addressing the psychological aspects, such as concerns about appearance and the potential for future dental procedures, can significantly improve patient satisfaction and adherence to the treatment plan. Ultimately, a holistic, patient-centered approach fosters a sense of trust and ensures that the patient’s needs are met both clinically and emotionally.

## 4. Conclusions

The effective management of complex dental trauma cases involving multiple permanent teeth requires a comprehensive, interdisciplinary approach, necessitating seamless coordination among various dental specialists. The timely integration of orthodontic, endodontic, and other relevant interventions is crucial for achieving optimal outcomes. Early and well-coordinated treatment is essential, as the precise timing of each intervention can significantly influence the long-term prognosis, ensuring the preservation of both function and aesthetics. Such a collaborative, multidisciplinary approach not only addresses the immediate clinical needs but also mitigates the risk of complications, fostering a more favorable and lasting result for the patient.

## Data Availability

Data supporting the findings of this case report are not publicly available due to patient privacy and ethical restrictions. All relevant information is included within the article.
